# Report of the 5th International Symposium on Frontiers in Molecular Science (ISFMS 2025)

**DOI:** 10.3390/ijms26189239

**Published:** 2025-09-22

**Authors:** Yoshinori Marunaka, Antonello Merlino, Maria Hrmova, Ye Chun Ruan, Atsushi Shiozaki, Masayuki Takahashi, Yusaku Iwasaki

**Affiliations:** 1 Medical Research Institute, Kyoto Industrial Health Association, Kyoto 604-8472, Japan; 2 Graduate School of Medical Science, Kyoto Prefectural University of Medicine, Kyoto 602-8566, Japan; 3 Research Organization of Science and Technology, Ritsumeikan University, Kusatsu 525-8577, Japan; 4 Department of Chemical Sciences, University of Naples Federico II, 80126 Naples, Italy; antonello.merlino@unina.it; 5 School of Agriculture, Food and Wine, and Waite Research Institute, Faculty of Sciences, Engineering and Technology, University of Adelaide, Glen Osmond, Adelaide, SA 5064, Australia; maria.hrmova@adelaide.edu.au; 6 Department of Biomedical Engineering, The Hong Kong Polytechnic University, Hong Kong SAR, China; sharon.yc.ruan@polyu.edu.hk; 7 Division of Digestive Surgery, Department of Surgery, Kyoto Prefectural University of Medicine, Kyoto 602-8566, Japan; shiozaki@koto.kpu-m.ac.jp; 8 School of Life Science and Technology, Tokyo Institute of Technology, Tokyo 152-8550, Japan; takahashi.m@kpu.ac.jp; 9 Laboratory of Animal Science, Graduate School of Life and Environmental Sciences, Kyoto Prefectural University, Kyoto 606-8522, Japan; ysk-iwasaki@kpu.ac.jp

**Keywords:** protein structure, molecular dynamics, enzymes, membrane proteins, cancer target proteins, drug design, drug resistance, physiological functions, organ interactions

## Abstract

The 5th International Symposium on Frontiers in Molecular Science was held on 26–29 August 2025 in Kyoto (Japan), with the support of Kyoto Prefectural University and Kyoto Prefectural University of Medicine. It is evident that the event has proven to be significant, showcasing presentations of pioneering research achievements by internationally renowned researchers and fostering numerous stimulating discussions. The symposium’s objective was to identify and select key research themes within the domain of molecular science. Three plenary lecturers and numerous researchers of outstanding merit were invited by chairs to deliver keynote and invited lectures across six fields: S1. Protein Structure and Molecular Dynamics; S2. Enzymes; S3. Membrane Proteins; S4. Cancer Target Proteins; S5. Drug Design and Solution to Drug Resistance Problem; S6. Physiological Functions of Proteins and Organ Interactions. A total of 185 scientists from 31 countries/regions participated in the symposium with 139 presentations. We would like to express our sincere gratitude to the 31 members of the Scientific Committee and the seven members of the Local Organizing Committee who contributed to enhancing the quality of this symposium, ensuring its smooth operation, and dedicating considerable effort to the selection of each award.

## 1. Introduction

The 5th International Symposium on Frontiers in Molecular Science was held on 26–29 August 2025 in Kyoto (Japan), with the support of Kyoto Prefectural University and Kyoto Prefectural University of Medicine (Japan). We are confident that this symposium has proven to be an extremely meaningful event, featuring the presentations of cutting-edge research achievements by world-leading researchers and fostering numerous lively discussions. This symposium selected key research themes within the fields of molecular science. Three plenary lecturers were invited, as well as numerous outstanding researchers, to deliver keynote and invited lectures across six fields: The plenary lecturers were presented by Prof. Dr. Harry Brumer (University of British Columbia, Canada), Prof. Dr. Hideki Sakai (University of Toyama, Japan), and Prof. Dr. Akiyuki Taruno (Kyoto Prefectural University of Medicine, Japan). Further, 14 keynote and 10 invited speakers were welcomed, and 27 oral and 85 poster presentations were delivered in six scientific sessions: S1. Protein Structure and Molecular Dynamics; S2. Enzymes; S3. Membrane Proteins; S4. Cancer Target Proteins; S5. Drug Design and Solution to Drug Resistance Problem; S6. Physiological Functions of Proteins and Organ Interactions. More than 185 scientists attended the symposium from 31 countries/regions with 139 presentations detailed above. We greatly appreciate the contributions from 31 Scientific Committee Members and seven Local Organizing Committee Members who enhanced the quality of the symposium, enabled smooth operation, and devoted considerable effort to the selection of awards.

## 2. Session Summary

In addition to three plenary lectures, we organized six sessions: S1. Protein Structure and Molecular Dynamics, organized by Prof. Dr. Antonello Merlino, University of Naples Federico II, Italy; S2. Enzymes organized by Prof. Dr. Maria Hrmova, University of Adelaide, Australia; S3. Membrane Proteins organized by Prof. Dr. Sharon Y.C. Ruan, The Hong Kong Polytechnic University, Hong Kong, China, and Prof. Dr. Billy K.C. Chow, The University of Hong Kong, Hong Kong, China; S4. Cancer Target Proteins organized by Prof. Dr. Atsushi Shiozaki, Kyoto Prefectural University of Medicine, Japan, and Prof. Dr. Yoshinori Marunaka, Kyoto Industrial Health Association, Japan, Kyoto Prefectural University of Medicine, Japan, Ritsumeikan University, Japan; S5. Drug Design and Solution to Drug Resistance Problem, organized by Dr. Masayuki Takahashi, Kyoto Prefectural University, Japan, and Institute of Science Tokyo, Japan; S6. Physiological Functions of Proteins and Organ Interactions, organized by Prof. Dr. Yusaku Iwasaki, Kyoto Prefectural University, Japan, and Prof. Dr. Yoshinori Marunaka, Kyoto Industrial Health Association, Japan, Kyoto Prefectural University of Medicine, Japan, Ritsumeikan University, Japan We conclude that all presenters, including keynote and invited speakers, and poster contributors, showcased valuable cutting-edge research in molecular science, making this symposium a worthwhile occasion. 

### 2.1. Session 1: “Protein Structure and Molecular Dynamics”

Session 1, entitled “Protein Structure and Molecular Dynamics”, focused on new frontiers in protein structure research. This part of the Conference began with Prof. Antonello Merlino, who chaired the session. Antonello Merlino is a full professor of Physical Chemistry at the University of Naples Federico II, Naples, Italy. He spoke about protein metalation [1,2,3], the process through which metal compounds react with proteins to form metal–protein adducts, where the metals generally directly coordinate protein residue side chains, providing examples of structures of adducts formed upon reaction of vanadium-based drugs and proteins [4,5].

The second keynote speaker was Dr. Luigi Vitagliano, Research Director at the Institute of Biostructures and Bioimaging of the Italian National Research Council, Naples, Italy. He presented on structure–function relationships in KCTDs [6], which are proteins containing the potassium channel tetramerization domain involved in many physiological and pathological processes, including the development of cancer, genetic, and neurodegenerative diseases. Combining structure prediction by AlphaFold and X-ray crystallography, he discovered unexpected similarities between different members of the KCTD family, also providing structural bases for the recognition of their biological partners [7,8,9].

This speech was followed by that of Dr. Faisal Koua from European XFEL GmbH, Germany, who described room temperature and cryogenic ultra-high-resolution structures (0.95–0.98 Å resolution) of a recombinant high-potential iron sulfur protein (HiPIP), a model metalloprotein with a single iron–sulfur cluster [10], solved by using ultra-short X-ray free electron laser pulses at megahertz repetition rate.

The following oral presenter was Dr. Ayub Hareed (University of Manchester, Singapore), who reported a new generalized machine-learning-based method that is able to back-map any protein and was validated using several large proteins, like immunoglobulin E and SARS-CoV-2 spike protein, typically used in coarse-grained applications [11].

The session went on with Xiakun Chu (Hong Kong University of Science and Technology, China), who gave a presentation on his data on the use of coarse-grained modeling to study the effect of transcription factors on enhancer–promoter interaction [12,13]. His talk highlights the critical role of chromatin dynamics in genome organization and regulation.

The last keynote speaker of this section was Prof. Victor Muñoz, Director of the NSF Center for Cellular and Biomolecular Machines and Professor of Bioengineering at the University of California, Merced, USA. Prof. Munoz summarized his works on the kinetic, thermodynamic, structural, and functional analysis of the simplest possible protein folds (fold archetypes) and their intimate connection with intrinsic disorder and functional dynamics [14,15,16].

This speech was followed by Dr. Giarita Ferraro, from the University of Naples Federico II, Naples, Italy, who described the use of proteins as metallodrug delivery systems [17], focusing on the structure–function relationship of metallodrug-encapsulated ferritin nanocages [18].

Next, Dr. Julien Hugo Mignon from the University of Namur, Belgium, showed how pH and ionic strength variations could affect the conformational and amyloidogenic landscapes of the intrinsically disordered double PHD finger 3 (DPF3) protein, investigating two DPF3 isoforms by a combination of spectroscopy, microscopy, and molecular dynamics simulations [19,20,21].

At this stage, two invited speakers were introduced: Prof. Edward D’Antonio, Full Professor of Biochemistry at the University of South Carolina Beaufort, USA, and Prof. Jean-Didier Maréchal, Full Professor of Physical Chemistry at the Autonomous University of Barcelona, Spain. Prof. D’Antonio studied derivatives of the antitrypanosomal molecule gossypol, which can inhibit glucose kinases of *Trypanosomal cruzi* and *Trypanosomal brucei*. He showed molecular docking studies able to reveal the mechanism of interaction of these potential drugs with the investigated kinases, and biochemical assays that suggest a therapeutic window for these molecules [22,23].

Prof. Marechal showed how the use of computational tools could be of help in understanding the protein metalation process. He showed his contribution to the development of software packages that can help the prediction of metal binding sites and the description of metal/protein interactions at the molecular level, providing examples of multiscale molecular modeling of metalation processes in many different proteins [24,25,26,27].

The last two speakers of the session were Dr. Florian Kongoli and Dr. Joseph Davis, from FLOGEN Technologies Inc., Montreal, QC, Canada, platinum sponsor of the meeting. Dr. Florian Kongoli showed the FLOGEN sustainability framework and its application in science and technology, and his point of view on sustainability in the field of drug development as the Executive President (CEO) of the company. Dr. Joseph Davis presented a unified theory of neurodegeneration pathogenesis, which establishes that the regulation of axon and dendrite-specific 4E-BP2 deamidation rates controls the occurrence and progression of neurodegenerative diseases [28,29].

### 2.2. Session 2: “Enzymes”

The “Enzymes” session was focused on the original and impactful research that showcased new aspects of enzyme kinetics and catalysis. The gender-balanced group of six distinguished and three emerging scientists from eight countries (Australia, Canada, China, Denmark, Japan, the Russian Federation, Spain, Thailand) presented the enzymology field as an exciting discipline that inspires bioengineering efforts to create a sustainable bioeconomy and play a fundamental role in life sciences and medicine.

This session was also open to other core areas, such as enzyme and chemical engineering, biofuel cells, and medical applications, to reveal new frontiers for the enzymology field. The key techniques in the presented research were X-ray crystallography, nuclear magnetic resonance spectroscopy, and computational modeling, including docking and molecular dynamics simulations. Other approaches included enzyme kinetics, microscopy, evolutionary analyses, metabolic profiling, molecular biology, and other biochemical, biophysical, and integrated approaches.

The first presenter, the chair of the “Enzymes” session and the keynote speaker, Professor Maria Hrmova (University of Adelaide, Australia), opened the session. She reported on the discovery of substrate–product processive catalytic mechanism in the plant Family-3 exo-hydrolases [30]. She highlighted the importance of this newly discovered mechanism for the degradation of polymeric carbohydrates by enzymes with a closed active site pocket [30]. She further described nanoscale reactant movements and defined reactant trajectories [31] and dynamics of water molecules [32], which play pivotal roles in the binding and conformational behavior of isomeric β-d-glucoside substrates.

The second keynote speaker, Professor Leila Lo Leggio (University of Copenhagen, Denmark), revealed the function of lytic copper-containing polysaccharide monooxygenases, which conduct an oxidative degradation of recalcitrant cellulose substrates [33]. She concluded that while the initial impetus for these enzymes came from biotechnological applications, such as bioethanol production, there are other diverse roles of these enzymes in nature [34].

The first invited speaker, Associate Professor Masahiro Nakajima (Tokyo University of Science, Japan), defined the mechanism of bacterial cyclic β-1,2-d-glucans synthesis and degradation [35]. His research highlighted phylogenetic, functional, and structural relationships in bacterial enzymes and how these relationships relate to plant pathogenicity [36]. Although these glycoside hydrolases also play key roles in symbiosis and osmoregulation.

The focus of the second invited speaker, Associate Professor Laura Masgrau (Universitat Autònoma de Barcelona, Spain), was on carbohydrate-processing and oxidative enzymes [30,31,32,37]. In her presentation, she explained how these enzymes modulate substrate specificity, affinity, inhibition, and protein–carbohydrate interactions, and how biomedicine and biotechnology utilize this knowledge [38].

The last (third) invited speaker, Professor Antoni Planas (Universitat Ramon Llull, Spain), reported on the directed evolution and re-engineering of bacterial, yeast, and microalgal glycosidases, glycosyltransferases, and carbohydrate esterases [39]. These enzymes are involved in the depolymerisation and deacetylation of chitin substrates, which operate as antifungal agents in crop protection [40].

Lastly, Professor Harry Brumer (University of British Columbia, Canada) delivered an inspiring plenary presentation focused on the application of enzymes and interlinked fundamental research in carbohydrate chemistry and enzymology with human health [41,42]. His data provided insights into the molecular basis of xyloglucan and other polysaccharide metabolism in human gut microbiota, to improve the nutrition and health of humans and animals [43].

In addition to experienced senior presenters in the “Enzymes” session, three young investigators, Zhiqi Cong (Chinese Academy of Sciences, China), Piyanut Pinyou (Suranaree University of Technology, Thailand), and Anastasia Pometun (Lomonosov Moscow State University, Russian Federation), delivered enthusiastic contributions related to hydrogen peroxide tunnel engineering, bi-enzymatic biocatalytic systems, and structure-function of bacterial enzymes, respectively.

In summary, the speakers in the “Enzymes” session delivered fascinating and breakthrough concepts underlying enzyme catalytic mechanisms, including the roles of enzymes in chemical transformations and metabolic pathways in a variety of pro- and eukaryotic organisms.

### 2.3. Session 3: “Membrane Proteins”

Membrane proteins are embedded within or associated with the lipid bilayer of cellular membranes, which serve a wide range of critical functions, including acting as channels and transporters for ions and molecules, receptors for signaling molecules, enzymes catalyzing biochemical reactions, and anchors for cellular structure. Their dynamic roles are fundamental to processes such as signal transduction, cellular communication, metabolism, and the maintenance of homeostasis. Dysfunction of membrane proteins is implicated in a vast array of human diseases, including cystic fibrosis, metabolic disorders, cardiovascular diseases, neurological conditions, and many forms of cancer. Additionally, membrane proteins are major targets for therapeutic intervention and drug development. This session brings together recent advances in the study of membrane proteins, with a focus on their molecular mechanisms, regulatory pathways, and implications for human health and diseases [44].

Session Talks:

1. Protein Quality Control Mechanisms of CFTR as Therapeutic Targets for Cystic Fibrosis (Tsukasa Okiyoneda)

Cystic fibrosis transmembrane conductance regulator (CFTR) is a cAMP-activated Cl^−^ channel mostly expressed in epithelial cells, mutation of which results in cystic fibrosis, the most common genetic disease in Caucasians. Frequent mutations of CFTR, such as DF508, lead to misfolding of the CFTR protein, triggering its degradation by protein quality control systems in cells. In this talk, Prof Okiyoneda presented recent advances in the identification of specific ubiquitin ligases responsible for CFTR degradation, as well as efforts in developing these ubiquitin ligases as drug targets to enhance the efficacy of existing treatments for cystic fibrosis [45,46].

2. Novel Roles of Epithelial Ion Channels in Endocrine Functions (Sharon Y.C. Ruan)

This presentation expands the traditional view of CFTR and ENaC ion channels, showing that beyond their established roles in epithelial fluid transport, they are also expressed in endocrine cells. Using conditional knockout mouse models and advanced imaging and molecular techniques, the research demonstrates direct involvement of these channels in ovarian steroidogenesis, bone remodeling, and glucose metabolism, offering new insights into endocrine physiology and related disorders [47,48,49].

3. Voltage Dependence of G Protein-Coupled Receptors (Yair Ben-Chaim)

This talk explores the emerging concept that G protein-coupled receptors (GPCRs) exhibit voltage-dependent changes in ligand affinity and activity. Focusing on muscarinic receptors, the speaker describes the discovery of a voltage-sensing motif and discusses the broader implications of voltage sensitivity as a general property of GPCRs, shedding light on new aspects of cellular signaling [50,51].

4. Functional Analysis of HECT-type E3 Ligase HERC3 in ER-associated Degradation of CFTR Mutants (Yuka Kamada)

This research identifies HERC3 as a novel E3 ubiquitin ligase involved in the ER-associated degradation of misfolded CFTR, independent of previously known ligases RNF5 and RNF185. Using innovative real-time assays and binding studies, the team demonstrates that HERC3 selectively recognizes and facilitates degradation of CFTR domains exposed from the ER membrane, advancing our understanding of CFTR quality control and potential therapeutic targets for cystic fibrosis [52].

5. MT1-MMP: New Therapeutic Target of Metabolic Disorder (Hoi Leong Xavier Wong)

This presentation uncovers the role of MT1-MMP in regulating the GDF15-GFRAL signaling axis, which controls appetite and body weight, and in modulating insulin sensitivity. The findings reveal new mechanisms underlying satiety and insulin resistance, particularly in obesity and aging, and identify a promising herbal compound that targets these pathways. MT1-MMP emerges as a novel therapeutic target for metabolic disorders [53,54].

Overall, this session showcases innovative research at the intersection of membrane protein biology, protein degradation, and metabolic regulation, highlighting new therapeutic strategies for cystic fibrosis, endocrine disorders, and metabolic diseases.

### 2.4. Session 4: “Cancer Target Proteins”

This session featured a variety of presentations on novel therapeutic target proteins in cancer, highlighting advances from fundamental research to translational and clinical perspectives. The session was chaired by Prof. Dr. Yoshinori Marunaka and Prof. Dr. Atsushi Shiozaki.

The first speaker was Prof. Dr. Atsushi Shiozaki (Kyoto Prefectural University of Medicine, Japan), who presented his keynote lecture on Ion Channel Profiling and Therapeutic Targeting in Cancer Stem Cells of Digestive Malignancies. He reported on distinct ion channel expression profiles in cancer stem cells, highlighting TRPV2 as a promising therapeutic target. Furthermore, he introduced the ongoing TNAC clinical trial evaluating tranilast-based therapy for esophageal cancer.

This keynote lecture was followed by Prof. Dr. Masahiro Ohsawa (Nagoya City University, Japan), who gave a presentation on Application of Kampo Medicines for the Palliation of Cancer Cachexia. He discussed the scientific basis of Kampo medicines, explaining their effects on appetite stimulation, skeletal muscle protein maintenance, and suppression of pro-inflammatory cytokines, all of which contribute to alleviating cancer cachexia.

The next speaker was Dr. Hiroki Shimizu (Kyoto Prefectural University of Medicine, Japan), who was invited to present The Potential of Ion Channels as Therapeutic Targets in Gastrointestinal Cancers. He summarized experimental and clinical findings on chloride and calcium channels, emphasizing their potential as novel biomarkers and therapeutic targets.

Following this, Dr. Sitthinon Siripanthong (University of Leeds, UK) delivered his oral presentation entitled “Using Adhiron Technology to Develop PAK1-Selective Inhibitors for Targeted Cancer Treatment”. He described the identification of Adhirons capable of selectively inhibiting PAK1 and discussed their potential applications as novel cancer therapeutics.

The next oral presentation was given by Dr. Rafaèle Calvo Esposito (Université Libre de Bruxelles, Belgium), who reported on “New Insights in the Proteolytic Contribution of Ananas comosus Extract to Its in vitro Anti-Cancer Effects against Colon Cancers”. She demonstrated that bromelain induces loss of cell adhesion and disruption of ion homeostasis in colon cancer cells, elucidating its anti-cancer effects.

The following keynote lecture was presented by Prof. Dr. Yoichiro Isohama (Tokyo University of Science, Japan), who spoke on “Novel Functions of Aquaporins in Cancer and Their Potential as Therapeutic Targets”. He highlighted the roles of aquaporins in tumor cell migration, proliferation, and apoptosis, especially the antioxidant and anti-apoptotic effects of AQP5, suggesting their promise as novel therapeutic targets.

Prof. Dr. Akira Ikari (Gifu Pharmaceutical University, Japan) then presented “Inhibition of Proliferation of Colorectal Cancer Cells by Suppression of Claudin-14 Expression”. He reported that CLDN14 silencing suppressed cell proliferation via inhibition of the Rho kinase/ERK pathway, proposing CLDN14 as a therapeutic target in colorectal cancer.

The session then continued with Dr. Oskar Kruc (Jagiellonian University, Poland), who gave his oral presentation entitled “Design of Potent Elongated m-Terphenylpicolinamide Derivatives and Their Interactions with the PD-L1 Protein”. He introduced newly developed PD-L1 inhibitors with nanomolar affinity, which enhanced T cell activation and cytotoxicity against cancer cells.

The next oral presentation was given by Dr. Motoki Watanabe (Kyoto Prefectural University of Medicine, Japan), who presented “Identification of ANT2 as a Druggable Target for Endocrine-Resistant ERα-Positive Breast Cancer”. He described how ANT2 was identified as a novel druggable target using chemoproteomic approaches, and demonstrated that targeting ANT2 could overcome endocrine resistance, suggesting new therapeutic strategies for ERα-positive breast cancer.

The last speaker of the session was Dr. Kento Kurashima (Kyoto Prefectural University of Medicine, Japan), who delivered his invited lecture on “The Role of Ferroptosis in Gastrointestinal Cancers and Development of Novel Therapeutics Targeting Ferroptosis”. He discussed the mechanisms of ferroptosis involving ROS production and lipid peroxidation, underscoring its importance as a novel therapeutic strategy for digestive tract cancers.

This session covered a wide spectrum of molecular targets, including cancer stem cells, cancer cachexia, membrane transporters, cell death regulation, immune checkpoints, as well as the application of natural products and traditional medicines. The presentations demonstrated how fundamental research is being translated into clinical trials and novel therapeutic strategies and emphasized the importance of international collaboration for the future development of molecularly targeted cancer therapies.

### 2.5. Session 5: “Drug Design and Solution to Drug Resistance Problem”

The “Drug Design” session focused on new strategies in drug development, particularly for resolving the problem of drug resistance. Eight speakers, including young researchers, from six countries (China, Israel, Italy, Japan, the Philippines, and the Russian Federation), presented various strategies for drug development. Additionally, 12 posters from eight countries were exhibited.

The first presenter, keynote speaker Dr. Masayuki Takahashi (Kyoto Prefectural University and Institute of Science Tokyo, Japan), opened the session by introducing an overview. He reported that drug resistance causes many deaths in infectious diseases and cancer. He then explained several recent strategies to cost effectively and quickly develop new treatments: (1) structure-based drug design, (2) AI-assisted approaches for all steps of drug development, including prediction of possible toxicity of compounds, (3) use of oligonucleotides, peptides, and proteins as drugs to avoid complicated chemical synthesis, (4) regulation of translation by oligonucleotides binding to the target mRNA like siRNA, (5) development of drug carriers to protect and deliver the drug at the action site, (6) slowing down the resistance emergence by concomitant use of several drugs (combination therapy) or inhibiting recombination, (7) use of genetically modified model animals to more conveniently examine drug efficacy.

The second keynote speaker, Assistant Professor Alessio Nocentini (University of Florence, Italy), explained the development of multi-target drugs, the most elegant form of combination therapy, using modulators of carbonic anhydrase. Multi-target drugs offer advantages over traditional combination therapies, including improved pharmacokinetic properties, reduced risk of drug–drug interactions, and enhanced synergistic effects. Professor Alessio Nocentini has developed drugs using advanced structure-based methods. He demonstrated that multi-target drugs consistently outperform corresponding single-target agents and their combinations across multiple disease models, such as glaucoma, cancer, inflammation, infections, and neurodegenerative diseases.

The invited speaker, Professor Assaf Friedler (Hebrew University of Jerusalem, Israel), proposed the use of peptides to analyze protein–protein interactions and treat diseases. Protein–protein interactions mediate various biological processes and can be related to diseases. However, it is challenging to analyze and make them drug targets because the interactions can involve intrinsically disordered parts and are modulated by post-translational modifications, such as phosphorylations. Professor Assaf Friedler prepared peptide libraries with distinct multi-phosphorylation patterns to elucidate how multi-phosphorylation regulates the self-assembly and activity of disease-related proteins. The study enables him to design peptide drugs that target protein–protein interactions.

Other researchers presented the following topics through their oral presentations or posters: a search for new drug targets, molecular screening, molecular docking-based drug design, optimization of antibody drugs, and development of multi-target drugs and drug delivery systems. As new targets, the regulation of ubiquitination and autophagy, as well as the inhibition of biofilm synthesis, were proposed. Assay of plant products was also reported. Drugs to treat neurodegenerative diseases and cancers were mainly addressed.

The session was well-connected with other sessions. Several presentations from other sessions were tightly related to our session topics, demonstrating an organic interaction among sessions and the advantage of a multi-session symposium.

In summary, the speakers and the poster presenters in the “Drug Design and Solution to Drug Resistance Problem” session delivered exciting and breakthrough concepts and a variety of strategies for drug development. The session provided a valuable opportunity for participants to pursue their research projects with a broader perspective.

### 2.6. Session 6: “Physiological Functions of Proteins and Organ Interactions”

This session examined the wide-ranging functions of proteins, including intracellular proteins, membrane-bound proteins, and peptide hormones, with an emphasis on their roles in whole-body physiology. Presentations focused on the in vivo roles of these proteins and highlighted recent discoveries that underscore the importance of organ-to-organ networks in systemic regulation. Groundbreaking studies were introduced that link complex physiological functions to individual protein molecules. Particular attention was given to research showing how single proteins can regulate diverse physiological processes throughout the body. By addressing protein functions from the perspectives of integrative physiology—including physiology, endocrinology, and neuroscience—the session successfully connected molecular findings to the broader field of protein science.

The first speaker, keynote speaker Dr. Yusaku Iwasaki, Professor at the Graduate School of Life and Environmental Sciences, Kyoto Prefectural University, presented on “Gut-derived glucagon-like peptide-1 induces satiation beneficial for obesity prevention.” He explained that endogenous GLP-1 and GLP-1 receptor agonists used as anti-obesity drugs exert their anti-obesity effects through entirely different mechanisms.

The second keynote speaker was Dr. Lok-To Sham, Assistant Professor in the Department of Microbiology and Immunology at the National University of Singapore. He presented on “Profiling the Genetic Interaction Landscape in Bacteria.” In his talk, he introduced Dual Tn-seq, a method that combines random barcode transposon-site sequencing (RB Tn-seq) with the Cre-lox system, allowing large-scale parallel analysis of about 1.4 billion double mutants in *Streptococcus pneumoniae*. Using this approach, his team revealed numerous genetic interactions, identified new factors in established pathways, and uncovered potential cellular roles for 69 previously uncharacterized proteins.

The third invited speaker was Dr. Kazunari Miyamichi, a team leader at RIKEN BDR in Japan. He presented on “Labeled-Line Circuits of Molecularly Delineated Sympathetic Neurons for Selective Organ Regulation.” In his talk, he described how, by combining recent single-cell transcriptomic datasets with viral genetic tools in mice, his team identified molecularly distinct subtypes of spinal sympathetic preganglionic neurons and clarified their organ-specific functions.

The fourth invited speaker was Dr. Yuta Masuda, a Specially Appointed Assistant Professor at Kyoto Prefectural University, who presented “Gut hormone cholecystokinin drives oxytocin-dependent thermogenesis in brown adipose tissue via the vagal sensory nerve–brain axis.” In his talk, he clearly demonstrated the input, central, and output mechanisms through which the gut hormone CCK induces thermogenesis in brown adipose tissue.

In addition to these keynotes and invited speakers, four oral presenters also gave presentations: Dr. Ryusuke Yoshida from Okayama University, Japan; Dr. Takashi Nakahari from Ritsumeikan University BKC, Japan; Dr. Sharifah Zamiah Syed Abdul Kadir from University Malaya, Malaysia; and Dr. Yu-Kuo Chen from National Pingtung University of Science and Technology, Taiwan. Each of them introduced distinctive proteins and highlighted their physiological significance.

## 3. Concluding Remarks

It is expected that the molecular science research presented at the 5th International Symposium on Frontiers in Molecular Science will make a significant contribution to the future development of this field, both qualitatively and by broadening its scope.

This symposium served as an important forum for the exchange of ideas, enabling the participants to deepen their knowledge and understanding of diverse issues in molecular sciences. Three plenary lectures provided audiences with insights into the molecular mechanisms regarding the human gut microbiota-plant cell wall nexus, novel insights into intracellular P-type ATPase functions in diseases, and the discovery of channels and their biological functions. The 14 keynote lectures, ten invited speeches, 27 oral and 85 poster presentations contributed new insights into the molecular science research field by presenting cutting-edge research in various areas within the themes of six sessions.

The knowledge and insights gained from this symposium will inform future research directions and offer potential solutions to current and forthcoming molecular science challenges we face.

## Data Availability

No new data were created or analyzed in this study. Data sharing is not applicable to this article.

## References

[1] Merlino A. (2021). Recent advances in protein metalation: Structural studies. Chem. Commun..

[2] Merlino A., Marzo T., Messori L. (2017). Protein Metalation by Anticancer Metallodrugs: A joint ESI MS and XRD Investigative Strategy. Chemistry.

[3] Messori L., Merlino A. (2017). Protein metalation by metal-based drugs: X-ray crystallography and mass spectrometry studies. Chem. Commun..

[4] Ferraro G., Tito G., Sciortino G., Garribba E., Merlino A. (2023). Stabilization and Binding of [V_4_O_12_]^4−^ and Unprecedented [V_20_O_54_(NO_3_)]^n−^ to Lysozyme upon Loss of Ligands and Oxidation of the Potential Drug V^IV^O(acetylacetonato)_2_. Angew. Chem. Int. Ed. Engl..

[5] Tito G., Ferraro G., Pisanu F., Garribba E., Merlino A. (2024). Non-Covalent and Covalent Binding of New Mixed-Valence Cage-like Polyoxidovanadate Clusters to Lysozyme. Angew. Chem. Int. Ed. Engl..

[6] Canettieri G., Di Marcotullio L., Greco A., Coni S., Antonucci L., Infante P., Pietrosanti L., De Smaele E., Ferretti E., Miele E. (2010). Histone deacetylase and Cullin3-REN(KCTD11) ubiquitin ligase interplay regulates Hedgehog signalling through Gli acetylation. Nat. Cell Biol..

[7] Esposito L., Balasco N., Vitagliano L. (2022). Alphafold Predictions Provide Insights into the Structural Features of the Functional Oligomers of All Members of the KCTD Family. Int. J. Mol. Sci..

[8] Balasco N., Esposito L., Smaldone G., Salvatore M., Vitagliano L. (2024). A Comprehensive Analysis of the Structural Recognition between KCTD Proteins and Cullin 3. Int. J. Mol. Sci..

[9] Balasco N., Pirone L., Smaldone G., Di Gaetano S., Esposito L., Pedone E.M., Vitagliano L. (2014). Molecular recognition of Cullin3 by KCTDs: Insights from experimental and computational investigations. Biochim. Biophys. Acta.

[10] Hirano Y., Takeda K., Miki K. (2016). Charge-density analysis of an iron–sulfur protein at an ultra-high resolution of 0.48 Å. Nature.

[11] Noid W.G. (2013). Perspective: Coarse-grained models for biomolecular systems. J. Chem. Phys..

[12] Zhu T., Li C., Chu X. (2025). Transcriptional Condensates Encode a “Golden Mean” to Optimize Enhancer-Promoter Communication across Genomic Distances. bioRxiv.

[13] Zhu T., Li C., Chu X. (2024). Fluctuating Chromatin Facilitates Enhancer–Promoter Communication by Regulating Transcriptional Clustering Dynamics. J. Phys. Chem. Lett..

[14] De Sancho D., Muñoz V. (2011). Integrated prediction of protein folding and unfolding rates from only size and structural class. Phys. Chem. Chem. Phys..

[15] Ghosh C., Nagpal S., Muñoz V. (2024). Molecular simulations integrated with experiments for probing the interaction dynamics and binding mechanisms of intrinsically disordered proteins. Curr. Opin. Struct. Biol..

[16] Luong T.D.N., Nagpal S., Sadqi M., Muñoz V. (2022). A modular approach to map out the conformational landscapes of unbound intrinsically disordered proteins. Proc. Natl. Acad. Sci. USA.

[17] Hong S., Choi D.W., Kim H.N., Park C.G., Lee W., Park H.H. (2020). Protein-Based Nanoparticles as Drug Delivery Systems. Pharmaceutics.

[18] Monti D.M., Ferraro G., Merlino A. (2019). Ferritin-based anticancer metallodrug delivery: Crystallographic, analytical and cytotoxicity studies. Nanomed. Nanotechnol. Biol. Med..

[19] Mignon J., Mottet D., Leyder T., Uversky V.N., Perpète E.A., Michaux C. (2022). Structural characterisation of amyloidogenic intrinsically disordered zinc finger protein isoforms DPF3b and DPF3a. Int. J. Biol. Macromol..

[20] Mignon J., Mottet D., Verrillo G., Matagne A., Perpète E.A., Michaux C. (2021). Revealing Intrinsic Disorder and Aggregation Properties of the DPF3a Zinc Finger Protein. ACS Omega.

[21] Leyder T., Mignon J., Mottet D., Michaux C. (2022). Unveiling the Metal-Dependent Aggregation Properties of the C-terminal Region of Amyloidogenic Intrinsically Disordered Protein Isoforms DPF3b and DPF3a. Int. J. Mol. Sci..

[22] Goyzueta-Mamani L.D., Pagliara Lage D., Barazorda-Ccahuana H.L., Paco-Chipana M., Candia-Puma M.A., Davila-Del-Carpio G., Sobreira Galdino A., Machado-de-Avila R.A., Giunchetti R.C., D’Antonio E.L. (2025). Exploring the Potential of Malvidin and Echiodinin as Probable Antileishmanial Agents Through In Silico Analysis and In Vitro Efficacy. Molecules.

[23] Green S.B., Lanier RJJr Carey S.M., Morgan D.R., Gracz H., Sherman J., Rodriguez A., D’Antonio E.L. (2021). Synthesis, biochemical, and biological evaluation of C2 linkage derivatives of amino sugars, inhibitors of glucokinase from *Trypanosoma cruzi*. Bioorganic Med. Chem. Lett..

[24] Rodríguez-Guerra Pedregal J., Sciortino G., Guasp J., Municoy M., Maréchal J.D. (2017). GaudiMM: A modular multi-objective platform for molecular modeling. J. Comput. Chem..

[25] Sciortino G., Garribba E., Maréchal J.D. (2019). Validation and Applications of Protein-Ligand Docking Approaches Improved for Metalloligands with Multiple Vacant Sites. Inorg. Chem..

[26] Alemany-Chavarria M., Rodríguez-Guerra J., Maréchal J.D. (2023). TALAIA: A 3D visual dictionary for protein structures. Bioinformatics.

[27] Fernández-Díaz R., Roldán-Martín L., Sodupe M., Sánchez-Aparicio J.E., Maréchal J.D. (2025). BioBrigit, a Hybrid Machine Learning and Knowledge-Based Approach to Model Metal Pathways in Proteins: Application to a Dicopper Tyrosinase. ACS Omega.

[28] Joseph D. (2024). The Fundamental Neurobiological Mechanism of Oxidative Stress-Related 4E-BP2 Protein Deamidation. Int. J. Mol. Sci..

[29] Joseph D. (2025). The Unified Theory of Neurodegeneration Pathogenesis Based on Axon Deamidation. Int. J. Mol. Sci..

[30] Streltsov V.A., Luang S., Peisley A., Varghese J.N., Ketudat Cairns J.R., Fort S., Hijnen M., Tvaroška I., Ardá A., Hrmova M. (2019). Discovery of processive catalysis by an exo-hydrolase with a pocket-shaped active site. Nat. Commun..

[31] Luang S., Fernández-Luengo X., Nin-Hill A., Streltsov V.A., Schwerdt J.G., Alonso-Gil S., Ketudat Cairns J.R., Pradeau S., Fort S., Hrmova M. (2022). The evolutionary advantage of an aromatic clamp in plant family 3 glycoside exo-hydrolases. Nat. Commun..

[32] Luang S., Fernández-Luengo X., Streltsov V.A., Maréchal J.D., Masgrau L., Hrmova M. (2025). The structure and dynamics of water molecule networks underlie catalytic efficiency in a glycoside exo-hydrolase. Commun. Biol..

[33] Sun P., Huang Z., Banerjee S., Kadowaki M.A.S., Veersma R.J., Magri S., Hilgers R., Muderspach S.J., Laurent C.V.F.P., Lo Leggio L. (2023). AA16 Oxidoreductases boost cellulose-active AA9 lytic polysaccharide monooxygenases from *Myceliophthora thermophila*. ACS Catal..

[34] Zong Z., Mazurkewich S., Pereira C.S., Fu H., Cai W., Shao X., Skaf M.S., Larsbrink J., Lo Leggio L. (2022). Mechanism and biomass association of glucuronoyl esterase: An α/β hydrolase with potential in biomass conversion. Nat. Commun..

[35] Nakazawa Y., Kageyama M., Matsuzawa T., Liang Z., Kobayashi K., Shimizu H., Maeda K., Masuhiro M., Motouchi S., Nakajima M. (2025). Structure and function of a β-1,2-galactosidase from *Bacteroides xylanisolvens*, an intestinal bacterium. Commun. Biol..

[36] Motouchi S., Komba S., Nakai H., Nakajima M. (2024). Discovery of anomer-inverting transglycosylase: Cyclic glucohexadecaose-producing enzyme from *Xanthomonas*, a phytopathogen. J. Am. Chem. Soc..

[37] Mendoza F., Gómez H., Lluch J.M., Masgrau L. (2016). α1,4-N-Acetylhexosaminyltransferase EXTL2: The missing link for understanding glycosidic bond biosynthesis with retention of configuration. ACS Catal..

[38] Mendoza F., Masgrau L. (2021). Computational modeling of carbohydrate processing enzymes reactions. Curr. Opin. Chem. Biol..

[39] Guidi C., Biarnés X., Planas A., De Mey M. (2024). Expanding the chitin oligosaccharide portfolio by engineering nodc chitin synthases in *Escherichia coli*. Curr. Res. Biotechnol..

[40] Pascual S., Planas A. (2021). Carbohydrate de-n-acetylases acting on structural polysaccharides and glycoconjugates. Curr. Opin. Chem. Biol..

[41] Panwar D., Briggs J., Fraser A.S.C., Stewart W.A., Brumer H. (2025). Transcriptional delineation of polysaccharide utilization loci in the human gut commensal *Segatella copri* DSM18205 and co-culture with exemplar Bacteroides species on dietary plant glycans. Appl. Environ. Microbiol..

[42] Golisch B., Cordeiro R.L., Fraser A.S.C., Briggs J., Stewart W.A., Van Petegem F., Brumer H. (2024). The molecular basis of cereal mixed-linkage β-glucan utilization by the human gut bacterium *Segatella copri*. J. Biol. Chem..

[43] Ma W.J., Wang C., Kothandapani J., Luzentales-Simpson M., Menzies S.C., Bescucci D.M., Lange M.E., Fraser A.S.C., Gusse J.F., Brumer H. (2025). Bespoke plant glycoconjugates for gut microbiota-mediated drug targeting. Science.

[44] Jelokhani-Niaraki M. (2022). Membrane Proteins: Structure, Function and Motion. Int. J. Mol. Sci..

[45] Taniguchi S., Fukuda R., Okiyoneda T. (2023). The multiple ubiquitination mechanisms in CFTR peripheral quality control. Biochem. Soc. Trans..

[46] Kamada Y., Fukuda R., Okiyoneda T. (2019). ELISA Based Protein Ubiquitylation Measurement. Bio Protoc..

[47] Chen Z., Xu J., Hu P., Du W., Chen J., Zhang X., Zhou W., Gao J., Zhang Y., Dai B. (2025). Defective Cystic Fibrosis Transmembrane Conductance Regulator Accelerates Skeletal Muscle Aging by Impairing Autophagy/Myogenesis. J. Cachexia Sarcopenia Muscle.

[48] Ma X., Xu R., Chen J., Wang S., Hu P., Wu Y., Que Y., Du W., Cai X., Chen H. (2024). The epithelial Na(+) channel (ENaC) in ovarian granulosa cells modulates Ca(2+) mobilization and gonadotrophin signaling for estrogen homeostasis and female fertility. Cell Commun. Signal..

[49] Wu Y., Que Y., Chen J., Sun L., Guo J., Ruan Y.C. (2023). CFTR Modulates Hypothalamic Neuron Excitability to Maintain Female Cycle. Int. J. Mol. Sci..

[50] Rozenfeld E., Tauber M., Ben-Chaim Y., Parnas M. (2021). GPCR voltage dependence controls neuronal plasticity and behavior. Nat. Commun..

[51] Tauber M., Ben-Chaim Y. (2024). Voltage Sensors Embedded in G Protein-Coupled Receptors. Int. J. Mol. Sci..

[52] Kamada Y., Ohnishi Y., Nakashima C., Fujii A., Terakawa M., Hamano I., Nakayamada U., Katoh S., Hirata N., Tateishi H. (2024). HERC3 facilitates ERAD of select membrane proteins by recognizing membrane-spanning domains. J. Cell Biol..

[53] Guo X., Asthana P., Zhai L., Cheng K.W., Gurung S., Huang J., Wu J., Zhang Y., Mahato A.K., Saarma M. (2024). Artesunate treats obesity in male mice and non-human primates through GDF15/GFRAL signalling axis. Nat. Commun..

[54] Guo X., Asthana P., Gurung S., Zhang S., Wong S.K.K., Fallah S., Chow C.F.W., Che S., Zhai L., Wang Z. (2022). Regulation of age-associated insulin resistance by MT1-MMP-mediated cleavage of insulin receptor. Nat. Commun..

